# Detecting Dementia Using Lexical Analysis: Terry Pratchett’s Discworld Tells a More Personal Story

**DOI:** 10.3390/brainsci16010094

**Published:** 2026-01-16

**Authors:** Melody Pattison, Ahmet Begde, Thomas D. W. Wilcockson

**Affiliations:** 1 School of English, Communication and Philosophy, Cardiff University, Cardiff CF10 3XB, UK; 2 School of Sport, Exercise and Health Sciences, Loughborough University, Loughborough LE11 3TU, UK; 3 Department of Psychiatry, Oxford University, Oxford OX3 7JX, UK

**Keywords:** dementia, Alzheimer’s disease, linguistics, lexical analysis, Terry Pratchett

## Abstract

**Background/Objectives**: Dementia, characterised by cognitive decline, significantly impacts language abilities. While the risk of dementia increases with age, it often manifests years before clinical diagnosis. Identifying early warning signs is crucial for timely intervention. Previous research has demonstrated that changes in language, such as reduced vocabulary diversity and simpler sentence structures, may be observed in individuals with dementia. This study investigates the potential of linguistic analysis to detect early signs of cognitive decline by examining the writing of Sir Terry Pratchett, a renowned author diagnosed with Posterior Cortical Atrophy (PCA), typically a form of dementia caused by Alzheimer’s disease. **Methods:** This study analysed 33 Discworld novels by Terry Pratchett, comparing linguistic features before and after a potential turning point identified through analysis of adjective type-token ratios (TTR). **Results:** A significant decrease in lexical diversity (TTR) was observed for nouns and adjectives in later works. Total wordcount increased, while lexical diversity decreased, suggesting a shift towards simpler language. This shift coincided with a decrease in adjective TTR below a defined threshold, occurring approximately ten years before Pratchett’s formal diagnosis. **Conclusions:** These findings suggest that subtle changes in linguistic patterns, such as decreased lexical diversity, may precede clinical diagnosis of dementia by a considerable margin. This research highlights the potential of linguistic analysis as a valuable tool for early detection of cognitive decline. Further research is needed to validate these findings in larger cohorts and explore the specific linguistic markers associated with different types of dementia.

## 1. Introduction

Dementia is an umbrella term for a range of conditions involving neurodegeneration or other aetiologies that result in significant cognitive decline, the most common of which is Alzheimer’s disease [1]. The cognitive decline observed in people with Alzheimer’s disease involves the gradual accumulation of toxic amyloid-beta and tau proteins causing neuronal damage [2], so identifying those people with signs of Alzheimer’s-related cognitive decline as early as possible would enable interventions to be utilised to delay or even prevent some of the damage. People with dementia may first notice they have an issue when they experience increased episodes of confusion or issues with memory or language (e.g., [3]). However, Alzheimer’s pathology likely begins many years and perhaps decades before the onset of symptoms [4]. Indeed, research has shown that there are earlier warning signs of dementia which may be too subtle for a patient to be aware of, for example, problems with attention (e.g., [5]). Further, research suggests that it is currently possible to predict who will experience dementia 12 years prior to formal diagnosis [6]. Therefore, it may be possible to identify at-risk people before their cognitive decline worsens. Dementia has an effect on both speech and writing (e.g., [7,8]); therefore, measuring the first signs of decline in these functions may provide an early biomarker for dementia. In early-stage Alzheimer’s, researchers have observed impairments in both producing and understanding words and sentences [9]. Therefore, by looking for changes in how someone uses language, then this may provide an early warning sign for dementia. The complexity of sentence structure, as measured by factors like the number of clauses per utterance, decreases with age in both spoken and written language [10]. Older adults struggle more with complex sentence structures, such as those with left-branching clauses, compared to younger adults [11].

Overall, linguistic changes are to be expected as people age (e.g., [12]). However, these changes become more profound within people with cognitive decline (e.g., [13]). If a patient’s writing history is available, then linguistic analysis techniques could be used to supplement clinical assessments or as a standalone early detection tool. Recent studies have done exactly this by measuring individual writers’ publications over their careers to analyse how their language use has evolved. Garrard et al. [14] studied the works of Iris Murdoch, a renowned English author who was diagnosed with Alzheimer’s posthumously. Her final novel, published shortly before her diagnosis, is widely considered to exhibit signs of cognitive decline. While Garrard found minimal differences in overall structure and syntax, they observed significant and consistent variations in lexical diversity and word choice between the final book and control books from earlier in Murdoch’s career. These results provide evidence that Alzheimer’s may indeed be measured using linguistic analysis, specifically the number of unique word-types relative to the overall wordcount. Le et al. [15] explored this further by including additional authors, additional books, and improved analysis techniques. Le et al. analysed two authors believed to have Alzheimer’s disease during their careers, Iris Murdoch and Agatha Christie, as well as P.D. James to act as a control participant, who published until the age of 88 without experiencing evidence of cognitive decline. They included twenty of Murdoch’s twenty-six novels, published between ages 35 and 76, sixteen of Christie’s novels written between ages 28 and 82, and fifteen of the novels of P.D. James. They then produced an analysis of the novels at the lexical level, using a variety of measures, including vocabulary size, lexical repetition, lexical specificity, word-class deficits, and fillers. Type-token ratio (TTR: e.g., [16]) calculates the proportion of unique words to the total wordcount, and the word-type introduction rate (WTIR: e.g., [17]), which measures the rate at which new words are introduced in the text, calculated every 10,000 words. Regarding lexical repetition, while intentional repetition can be a stylistic device, an increasing rate of repeated words may suggest a limited vocabulary or difficulty accessing words. To examine this, they conducted two analyses: a global analysis and a local analysis. Lexical specificity is calculated by the frequency of indefinite nouns and high-frequency, low-imagery verbs in each text. A higher proportion of these generic words suggests lower overall lexical specificity. Word class deficit (WCD: e.g., [18]) is an analysis of the distribution of word classes across each text, examining both the total number of words and the number of unique words. This allows for identification of potential deficits or overreliance on specific word classes and to measure the vocabulary size of open classes. Filler words are a measure of the proportion of interjections and filler words. While these words often appear in dialogue, fiction authors strive for natural-sounding conversations. However, this measure may be influenced by stylistic choices rather than cognitive decline and should be interpreted with care. Le, et al. observed that P.D. James maintained stable linguistic diversity into their late 80s, whereas, for Iris Murdoch and Agatha Christie TTR and WTIR were associated with cognitive decline and a decline in vocabulary led to an increase in repetitions in content words, and a word-class deficit can be seen in noun-token proportion, with a compensatory increase in verb-token proportion. They also observed a deficit in noun tokens that is significantly correlated with a rise in verb and pronoun tokens. Syntactic-complexity results were also found to fluctuate in a relatively wider range. Interestingly, they also report that deficits in Murdoch’s writing appeared in Murdoch’s late 40s and early 50s, which suggests that language deficits are observed many years before a formal diagnosis and indicates that Alzheimer’s disease has a long preclinical period. Therefore, linguistic analysis would appear to show promise in identifying whether an author has experienced cognitive decline and may even indicate when the preclinical phase of dementia has begun.

The current research further explores the idea of using lexical analysis in dementia by studying the works of Sir Terry Pratchett. Terry Pratchett was an English author, humourist, and satirist, best known for his Discworld series of 41 comic fantasy novels published between 1983 and 2015. Terry Pratchett was diagnosed with Posterior Cortical Atrophy (PCA) in December 2007. This diagnosis came at a time when he was still actively writing and publishing his beloved Discworld series. Despite the challenges posed by his condition, Pratchett continued to write and advocate for dementia awareness until his passing in 2015. PCA is a rare form of Alzheimer’s disease that primarily affects visual processing and spatial awareness [19]: although note that 15% of PCA cases may be unrelated to AD pathology; see [20]. It affects areas in the back of the brain responsible for spatial perception, complex visual processing, spelling, and calculation [21]. PCA has also been found to be associated with word retrieval difficulties [22]. Given Terry Pratchett’s prolific writing career and the fact he continued writing after his diagnosis, a linguistic analysis of his novels could provide valuable insights into the potential early signs of cognitive decline. By comparing his earlier works to his later ones, particularly those written closer to his dementia diagnosis, it would be possible to identify subtle changes in linguistic patterns, such as decreased lexical diversity, and a decline in the use of specific word classes, such as nouns and adjectives, potentially preceding clinical diagnosis.

This study further explores the potential of lexical analysis as a tool for the early detection of cognitive decline by examining the writing of Sir Terry Pratchett, an author diagnosed with PCA. By analysing his Discworld series, it may be possible to identify subtle changes in linguistic patterns, specifically lexical diversity, that may have occurred prior to his formal diagnosis. Novels from the Discworld series were analysed to identify subtle changes in linguistic patterns, specifically lexical diversity as measured by TTR and Moving Average TTR (MATTR: e.g., [23]). The analysis focuses on whether these measures changed over time and their accuracy in predicting the linguistic changes associated with his diagnosis is evaluated. These findings may have implications for the use of lexical analysis in dementia research, highlighting its potential as a supplementary tool for early detection.

## 2. Materials and Methods

### 2.1. Materials

Lexical diversity was quantified across 33 of the 41 novels in the Discworld series. Eight titles were excluded from the analysis due to them being either shorter than the other full-length novels (*Eric, 1990; The Last Hero, 2001*), or because they are part of his titles for younger readers (*The Amazing Maurice and His Educated Rodents, 2001; The Wee Free Men, 2003; A Hat Full of Sky, 2004; Wintersmith, 2006; I Shall Wear Midnight, 2010; The Shepherds Crown, 2015*). These eight titles were excluded to ensure the validity of the lexical analysis. TTR is highly sensitive to text length; therefore, shorter works (e.g., *Eric*, which was originally published as an illustrated picture book) would yield artificially inflated diversity scores compared to full-length novels. Additionally, works written for younger readers were omitted because linguistic constraints and vocabulary choices for children differ fundamentally from those used in the main series. By focusing on a homogeneous corpus of adult-marketed, full-length novels, we ensured that observed changes reflect cognitive status rather than shifts in intended audience or format.

### 2.2. Data Extraction

The analysis focused on four major lexical word classes: nouns, verbs, adjectives, and adverbs. The corpus-analysis platform SketchEngine [24] was selected for data extraction because it supports large corpora, provides reliable part-of-speech tagging, and allows the retrieval of lemma-level frequency counts for specified word classes.

Plain-text versions of the 33 eligible novels were uploaded to SketchEngine. For each book, the number of unique lemmas in each word class was divided by the total number of tokens of that class, yielding a class-specific TTR. These measures provided an initial estimate of lexical diversity across the corpus.

To investigate potential effects of cognitive decline, novels were grouped according to publication relative to Pratchett’s PCA diagnosis in 2007: 29 books were classified as pre-diagnosis and 4 as post-diagnosis. Because TTR is sensitive to text length and unequal token counts across books, we additionally computed the moving-average type–token ratio (MATTR) with a fixed window size of 100 words. MATTR normalises diversity estimates across texts of different lengths and provides a more stable measure of lexical variation. These MATTR values were then included in subsequent statistical comparisons of pre- and post-diagnosis linguistic patterns.

MATTR is widely regarded as one of the most robust normalised diversity metrics, as it maintains sensitivity to lexical variation while minimising the length dependency that affects traditional TTR measures. Importantly, MATTR offers advantages over alternative normalisation procedures such as random sampling, fixed-sample TTR, or computationally intensive indices such as Measure of Textual Lexical Diversity (MTLD) or Hypergeometric Distribution (HD-D). Random sampling or fixed-sample TTR can introduce instability when texts differ markedly in style or local density of lexical items, while MTLD and HD-D, although informative, are more sensitive to distributional irregularities and may produce inflated variability when applied to long narrative texts. In contrast, MATTR computes TTR across overlapping windows, ensuring that all parts of a text contribute equally. This property makes MATTR particularly well suited for comparing long-form prose of variable length, such as the Discworld novels, and for detecting subtle changes in lexical choices over time. These MATTR values were therefore used in the statistical comparisons of pre- and post-diagnosis linguistic patterns.

### 2.3. Statistical Analyses

Descriptive statistics were calculated to summarise the key linguistic features used in Pratchett’s books. Independent *t*-tests were used to compare linguistic measures released before and after the dementia diagnosis. Linear regression analyses were conducted to investigate the relationship between various TTR types and age. Additionally, Receiver Operating Characteristic (ROC) curve analyses were performed to evaluate the accuracy of TTR measures in distinguishing between pre- and post-diagnosis phases. All statistical analyses were performed using SPSS (version 29), with the significance level set at *p* < 0.05.

## 3. Results

### 3.1. Pre- and Post-Dementia Diagnosis

The analysis included 33 books (see Table 1), with 29 books written before the dementia diagnosis and four books written after the diagnosis. Independent *t*-tests were conducted to compare linguistic features between these periods (see Table 2).

The analysis showed significant changes in several linguistic features following Pratchett’s dementia diagnosis. Books written after diagnosis showed a decrease in lexical diversity, as shown by significant lower type-token ratios for nouns (*p* = 0.044) and adjectives (*p* = 0.028). No significant changes were observed in the TTRs for verbs and adverbs, suggesting that some aspects of linguistic complexity remained stable after the diagnosis.

### 3.2. Decline in Lexical Diversity

**Figure 1:**
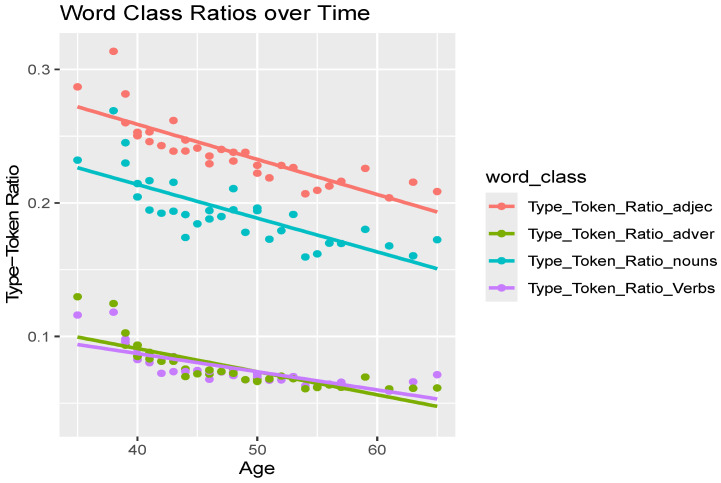
Type/token ratio over time in Pratchett’s Discworld books. Note, “age” represents Pratchett’s age when each book was published.

Figure 1 shows the changes in TTR across different word classes (adjectives, adverbs, nouns, and verbs) in Pratchett’s writing over time. All word classes demonstrated a declining trend in TTR with age, indicating a general decrease in lexical diversity. Linear regression analyses were conducted to examine the relationship between various TTRs and Pratchett’s age. All models showed significant predictive relationships (*p* < 0.001). The TTR for adjectives emerged as the strongest predictor (F(1,31) = 73.101, *p* < 0.001), followed by adverb TTR (F(1,31) = 53.694, *p* < 0.001), noun TTR (F(1,31) = 45.728, *p* < 0.001), and verb TTR (F(1,31) = 39.413, *p* < 0.001) also showed significant relationships with Pratchett’s age-related progression. These findings indicate that age-related progression is a significant predictor of the observed decline in lexical diversity.

### 3.3. Identification of Change Point

**Figure 2:**
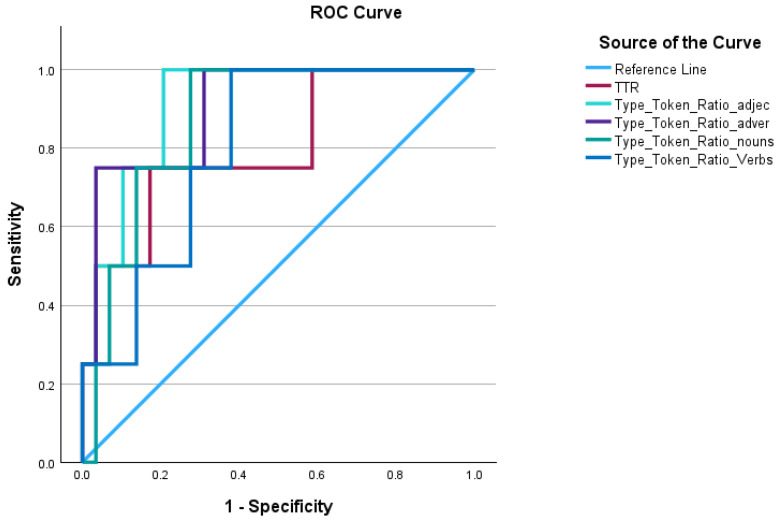
Receiver Operating Characteristic (ROC) curve analysis for the TTR variables.

Receiver Operating Characteristic (ROC) curve analysis was performed to assess the accuracy of various TTR measures in detecting linguistic changes associated with dementia (see Figure 2). All TTR measures demonstrated significant predictive ability (*p* < 0.05), with AUC values ranging from 0.80 to 0.91.

The TTR for adjectives showed the highest classification accuracy (AUC = 0.91, 95% CI: 0.80–1.02, *p* < 0.001: see Table 3), with a cut-off value of 0.227 showing 76% sensitivity and 100% specificity. This was followed by adverb TTR (AUC = 0.90, 95% CI: 0.76–1.04, *p* < 0.001), which demonstrated 96% sensitivity and 75% specificity at a cut-off of 0.062. Noun TTR also showed strong predictive performance (AUC = 0.87, 95% CI: 0.73–1.00, *p* < 0.001), with 86% sensitivity and 75% specificity at a cut-off of 0.182. Verb TTR show relatively lower but significant accuracy (AUC = 0.80, *p* = 0.001). Verb TTR achieved highest specificity (100%) but lower sensitivity (62%) at a cut-off of 0.072.

As TTR for adjectives showed the highest diagnostic accuracy, we can use the cut-off value of 0.227 to identify when in Pratchett’s writing his TTR for adjectives started to fall below this cut-off score. It was found that eleven of Pratchett’s works were found to have a TTR for adjectives lower than 0.227, with the earliest published being *The Last Continent* (Discworld 22), which was published in May 1998—9 years and 7 months before his formal diagnosis. All books published after this date were found to have a TTR for adjectives of less than 0.227 whilst all books published before this date were found to have a TTR for adjectives more than 0.227. The only outliers are Discworlds 23 (*Carpe Jugulum*) and 25 (*The Truth*), which both had scores of 0.228, which do not meet the cut-off despite being published after Discworld 22, but are only 0.001 outside the cut-off.

### 3.4. Pre- and Post-Change Point Analysis

Because Discworld 22 was the first book to fall below the TTR for adjectives cut-offs, independent *t*-tests were performed on pre and post Discworld 22 to compare linguistic features between these periods (see Table 4).

The analysis showed a decrease in lexical diversity, as shown by significant lower type-token ratios for nouns: *p* < 0.001; verbs: *p* < 0.001; adjectives: *p* < 0.001; and adverbs: *p* < 0.001. These results indicate overall that linguistic features differed on all aspects pre and post Discworld 22.

### 3.5. Moving Average Type Token Ratio

Finally, the total number of words per book was found to significantly differ pre (M = 87,197; SD = 14,372) and post (M = 116,152; SD = 12,303) book 22 (t(31) = 5.972; *p* < 0.001), which may affect TTR scores. Therefore, to control for this each TTR score was standardised by calculating the estimated moving average type token ratio (MATTR) with a window set at every 100 words. Independent *t*-tests were then performed on pre and post Discworld 22 books to compare linguistic features between these periods. It was found that even after controlling for word total, all MATTRs (noun, verb, adjective, adverb) differed significantly pre and post Discworld 22 (see Table 5). These results indicate that book word total did not account for the significant differences in TTRs.

## 4. Discussion

The current study aimed to explore the potential of lexical analysis as a tool for early detection of cognitive decline, specifically focusing on the case of Terry Pratchett and his diagnosis of PCA due to Alzheimer’s disease. Our analysis of Pratchett’s Discworld series revealed significant changes in linguistic patterns over time. The most notable finding was a significant decrease in lexical diversity, as measured by TTR, and then MATTR, for adjectives and nouns in Pratchett’s later works. This suggests a decline in vocabulary richness and a reliance on simpler language structures. While the overall TTR remained relatively stable, the decrease in lexical diversity within specific word classes indicates a subtle but significant change in linguistic style (see [15]). Our analysis of Pratchett’s works suggests that similar lexical changes could potentially also be observed in individuals with PCA due to Alzheimer’s disease.

The high predictive accuracy of TTR measures, particularly for adjectives, suggests that lexical analysis could be a valuable tool for early detection of cognitive decline. By identifying subtle changes in language use, it may be possible to detect preclinical changes in cognitive decline or even the onset of dementia years before a formal diagnosis. This early detection could enable timely interventions and potentially slow the progression of the disease. The analysis revealed a shift in Pratchett’s writing style following the publication of *The Last Continent* (Discworld 22). This book may indicate the point where the developing dementia pathology first becomes symptomatic, as it was the first to exhibit a TTR for adjectives below an AUC-established cut-off value of 0.227, a threshold with a high diagnostic accuracy for potentially detecting linguistic changes related to dementia in the Discworld series. To further investigate this shift, we compared linguistic features in books published before and after *The Last Continent*. The results revealed significant differences across various measures. Post-*Last Continent* books exhibited a significant decrease in lexical diversity across all word classes as measured by TTR. These findings suggest a shift in Pratchett’s writing style after this point. It should be noted that this book was published 9 years and 7 months before his formal diagnosis, indicating a long preclinical period prior to diagnosis. This observation is striking and highlights the potential for lexical analysis to identify subtle changes in writing style that may precede clinical diagnosis of dementia by a considerable margin.

The high classification accuracy observed in the ROC analysis (e.g., AUC = 0.91 for adjective TTR) provides robust evidence of a distinct shift in linguistic patterns following Book 22. However, it is important to distinguish between the intra-individual predictive power shown here and inter-individual clinical utility. In this study, the ROC curve functions as a tool to retrospectively identify the onset of a pathological trend within a single author’s long-term output. While these findings highlight the sensitivity of lexical diversity as a marker for preclinical cognitive decline, the specific cut-off values (such as the 0.227 threshold for adjectives) are calibrated to Pratchett’s unique baseline vocabulary and the specific genre requirements of the Discworld series. Therefore, these metrics do not serve as a universal screening tool for the general population. Instead, they demonstrate the potential for personalised, longitudinal linguistic monitoring—where a patient serves as their own control—to flag early signs of neurodegeneration that might be missed by standardised, cross-sectional cognitive assessments.

However, it is crucial to acknowledge the limitations of this study. First, the analysis was based on a single case study, limiting the generalisability of the findings. The results may be specific to Pratchett; therefore, further research is needed to validate these findings in a larger sample of individuals with dementia, including those with PCA. Relatedly, as this is a single-case study, without a control author, it remains possible that the decline in lexical diversity reflects natural ageing. But note that previous research [15] has found that healthy authors can maintain stable linguistic diversity into their late 80s, suggesting that the decline observed in Pratchett’s TTR is indicative of pathology rather than typical ageing. Second, a potential limitation of the analyses is that the 33 novels, produced over a 32-year career, may naturally be correlated and thus not strictly independent. While ignoring such dependencies can violate statistical assumptions and overstate significance, treating each work as a discrete “snapshot” of the author’s cognitive state is scientifically justified. This approach views each novel as a massive, distinct creative undertaking and aligns with established methodologies used to map cognitive decline in authors like Iris Murdoch and Agatha Christie [14,15]. Third, while PCA and Alzheimer’s disease share some common features and PCA is thought to be in most cases caused by Alzheimer’s disease (see [25]), they are distinct conditions with different clinical presentations [19]. It is possible that the linguistic markers of PCA may differ from those of Alzheimer’s disease. For example, PCA often causes severe reading impairments and “pure alexia,” which disrupts the writer’s ability to visually scan and review their own text. Writers unable to effectively read their drafts may struggle to detect unwanted lexical repetitions and potentially may contribute to the observed decline in lexical richness. Furthermore, several methodological limitations should be considered. The precise chronology of Pratchett’s writing is uncertain. We do not know the exact dates of writing for each book, whether he worked on multiple books concurrently, or if the publication order accurately reflects the writing order. These uncertainties could introduce potential biases into the analysis. Additionally, the observed changes in Pratchett’s writing style after his diagnosis may not solely be attributed to PCA. Factors such as reduced writing time due to the disease, potential collaboration with other writers, or significant editorial alterations could also have contributed to these changes. Finally, it is crucial to emphasise that the observed changes in Pratchett’s writing may not exclusively reflect cognitive decline due to PCA. Age-related changes in writing style are expected, and it is possible that some of the observed changes represent natural stylistic evolution rather than disease-related decline. Further, it is also worthy of note that Pratchett was writing with full knowledge of his condition and prognosis, which may have added a powerful psychological dimension to any biological influences on the language system. Therefore, we cannot wholly attribute changes in language due to Alzheimer’s disease pathology alone.

Despite these limitations, this study provides valuable insights into the potential of linguistic analysis as a tool for detecting early signs of cognitive decline. By analysing the works of Terry Pratchett, we have demonstrated how subtle changes in language use can be indicative of underlying cognitive impairment. Further research is needed to explore the full potential of linguistic analysis as a diagnostic tool for dementia, including the development of more sophisticated analytical methods and the investigation of larger and more diverse datasets. Future research could explore the use of computational linguistics and machine learning analysis techniques, to identify linguistic markers of cognitive decline. These techniques already show promise and are rapidly gaining attention in Alzheimer’s disease biomarker development (e.g., [26,27,28,29]). By developing more sensitive and accurate diagnostic tools, we may be able to improve early detection and intervention for dementia.

## 5. Conclusions

In conclusion, our study provides evidence that linguistic analysis can be a valuable tool for detecting early signs of cognitive decline. By analysing the works of Terry Pratchett, we have demonstrated how subtle changes in language use can be indicative of underlying cognitive impairment. The results also emphasise that language deficits may be observed many years before a formal diagnosis and indicate that Alzheimer’s disease has a long preclinical period—in the case of Terry Pratchett, potentially almost ten years. Further research is now needed to explore the full potential of linguistic analysis as a diagnostic tool for dementia.

## Figures and Tables

**Table 1:** Descriptive statistics for all the variables extracted from Pratchett’s Discworld books.

	Range	Minimum	Maximum	Mean	Std. Dev.
TTR Nouns	0.1096	0.1595	0.2691	0.1936	0.0251
TTR Verbs	0.0590	0.0592	0.1182	0.0763	0.0139
TTR Adjectives	0.1097	0.2038	0.3135	0.2379	0.0240
TTR Adverbs	0.0690	0.0607	0.1298	0.0771	0.0167
MATTR Nouns	0.1650	0.1558	0.3208	0.1884	0.0313
MATTR Verbs	0.0594	0.0591	0.1185	0.0761	0.0141
MATTR Adjectives	0.1097	0.2038	0.3135	0.2379	0.0240
MATTR Adverbs	0.0690	0.0607	0.1298	0.0771	0.0167

**Table 2:** Comparisons of Linguistic Features Pre- and Post-Dementia Diagnosis.

Linguistic Features	Pre-Diagnosis Mean (SD) (n = 29)	Post-Diagnosis Mean (SD) (n = 4)	*p*-Value
TTR Nouns	0.197 (0.025)	0.170 (0.008)	0.044 *
TTR Verbs	0.078 (0.014)	0.067 (0.005)	0.138
TTR Adjectives	0.241 (0.024)	0.213 (0.010)	0.028 *
TTR Adverbs	0.079 (0.017)	0.063 (0.004)	0.076

Note: * indicates statistical significance (*p* < 0.05).

**Table 3:** Area Under the Curve results for each TTR variable.

Measure	AUC (95% CI)	Cut-Off	Sensitivity	Specificity	*p*-Value
TTR Adjectives	0.91 (0.80–1.02)	0.227	0.76	1.00	<0.001 *
TTR Adverbs	0.90 (0.76–1.04)	0.062	0.96	0.75	<0.001 *
TTR Nouns	0.87 (0.73–1.00)	0.182	0.86	0.75	<0.001 *
TTR Verbs	0.80 (0.62–0.98)	0.072	0.62	1.00	0.001 *

Note: AUC = Area Under the Curve; CI = Confidence Interval; * indicates statistical significance (*p* < 0.05); TTR = Total-Type Token Ratio.

**Table 4:** Comparisons of Linguistic Features Pre- and Post-Discworld 22.

Linguistic Features	Pre-Discworld 22 Mean (SD) (n = 20)	Post-Discworld 22 Mean (SD) (n = 13)	*p*-Value
TTR Nouns	0.206 (0.024)	0.175 (0.013)	<0.001 *
TTR Verbs	0.083 (0.015)	0.067 (0.004)	<0.001 *
TTR Adjectives	0.251 (0.021)	0.217 (0.009)	<0.001 *
TTR Adverbs	0.085 (0.017)	0.065 (0.004)	<0.001 *

Note: * indicates statistical significance (*p* < 0.05).

**Table 5:** Comparisons of Standardised TTRs Pre- and Post-Discworld 22.

Linguistic Features	Pre-Discworld 22 Mean (SD) (n = 20)	Post-Discworld 22 Mean (SD) (n = 13)	*p*-Value
MATTR Nouns	0.1987 (0.0311)	0.1725 (0.0252)	0.008 *
MATTR Verbs	0.0823 (0.0148)	0.0665 (0.0375)	<0.001 *
MATTR Adjectives	0.2513 (0.0210)	0.2171 (0.0085)	<0.001 *
MATTR Adverbs	0.0851 (0.0648)	0.0648 (0.0037)	<0.001 *

Note: * indicates statistical significance (*p* < 0.05).

## Data Availability

Due to the nature of this research, copyright issues may restrict us from sharing data publicly. However, data and analysis code will be made available to individuals from the corresponding author upon request. Note that this project was not preregistered.
